# In Vivo Molecular Changes in the Retina of Patients With Multiple Sclerosis

**DOI:** 10.1167/iovs.62.6.11

**Published:** 2021-05-11

**Authors:** Salut Alba-Arbalat, Magi Andorra, Bernardo Sanchez-Dalmau, Anna Camos-Carreras, Marina Dotti-Boada, Irene Pulido-Valdeolivas, Sara Llufriu, Yolanda Blanco, Maria Sepulveda, Albert Saiz, Oscar Batet, Iker Bilbao, Iratxe Torre, Ivan Amat-Roldan, Elena H. Martinez-Lapiscina, Pablo Villoslada

**Affiliations:** 1 Center of Neuroimmunology, Institut d'Investigacions Biomediques August Pi Sunyer, Barcelona, Spain; 2 Department of Ophthalmology, Hospital Clinic, University of Barcelona, Barcelona, Spain; 3 Department of Neurology, Hospital Clinic, University of Barcelona, Barcelona, Spain; 4 Institut de Neurociències, University of Barcelona, Barcelona, Spain; 5 Expert Ymaging SL, Barcelona, Spain; 6 Stanford University, Stanford, California, United States

**Keywords:** molecular imaging, retina, multiple sclerosis, Raman spectroscopy

## Abstract

**Purpose:**

Raman spectroscopy allows molecular changes to be quantified in vivo from the tissues like the retina. Here we aimed to assess the metabolic changes in the retina of patients with multiple sclerosis (MS).

**Methods:**

We built a Raman spectroscopy prototype by connecting a scanning laser ophthalmoscope to a spectrophotometer. We defined the spectra of 10 molecules participating on energy supply, axon biology, or synaptic damage, which have been shown to be altered in the brain of patients with MS: cytochrome C, flavin adenine dinucleotide (FAD), nicotinamide adenine dinucleotide (NADH), *N*-acetyl-aspartate (NAA), excitotoxicity, glutamate, amyloid β (Aβ), τ and α-synuclein (SNCA), phosphatidyl-ethanolamine, and phosphatidyl-choline. We studied these molecules in a prospective cohort of patients with MS, either in the chronic phase or during relapses of acute optic neuritis (AON).

**Results:**

Significant changes to all these molecules were associated with age in healthy individuals. There was a significant decrease in NADH and a trend toward a decrease in NAA in patients with MS, as well as an increase in Aβ compared with healthy controls. Moreover, NADH and FAD increased over time in a longitudinal analysis of patients with MS, whereas Aβ diminished. In patients with acute retinal inflammation due to AON, there was a significant increase in FAD and a decrease in SNCA in the affected retina. Moreover, glutamate levels increased in the affected eyes after a 6-month follow-up.

**Conclusions:**

Alterations of molecules related to axonal degeneration are observed during neuroinflammation and show dynamic changes over time, suggesting progressive neurodegeneration.

The ability to monitor molecular changes in the central nervous system (CNS) in vivo is paramount to improve our understanding of disease pathogenesis or to identify new therapeutic targets, particularly in patients with acute or chronic diseases. At present, the molecular imaging technologies available to interrogate the CNS include positron emission tomography (PET), magnetic resonance spectroscopy (MRS), and near-infrared imaging (NIR), yet they each have several drawbacks. PET imaging has limited spatial and temporal resolution and requires specific radioligands, restricting the number of molecules that can be interrogated. MRS also has limited spatial and temporal resolution and is limited to just a few molecules. Finally, NIR also suffers from restricted spatial resolution and requires the use of specific fluorophores. Therefore, new technological solutions are required to obtain more comprehensive and higher-resolution assessment of the molecular changes in the CNS associated with disease. One potential solution is to take advantage of the potential of light-based technologies, such as Raman spectroscopy, and the accessibility of the retina as a part of the CNS.

Raman spectroscopy is a laser-based technique that identifies the chemical properties of tissues as it detects the molecular vibration frequencies that characterize molecular bonds and chemical structures.^1^ Raman spectroscopy has been successfully used to discriminate tumor-infiltrated tissues from noninfiltrated tissues in brain surgery based on the molecular differences in these tissues.^2^ Because the retina is a portion of the CNS that is directly accessible to laser-based technologies, Raman spectroscopy can be used to achieve molecular imaging of CNS and retina diseases in vivo.^3^ Like other gray matter structures, the retina exhibits both inflammation and neurodegeneration in many brain diseases.^4^ Indeed, we have previously shown in preclinical studies that Raman spectroscopy can detect in vitro relevant molecular changes in retinal inflammation, such an increase of immune mediators (lipoxygenase, inducible nitric oxide synthase, and TNFα), changes in molecules involved in energy production (cytochrome C [CytC], phenylalanine, and nicotinamide adenine dinucleotide [NADH]/NAD^+^), and a decrease of phosphatidylcholine (PC).^5^

Optical coherence tomography (OCT) studies have revealed that the retina is damaged in neuroinflammatory diseases, particularly during acute inflammation such as that occurring in acute optic neuritis (AON)^6^ or neuromyelitis optica (NMO),^7^ although chronic and progressive damage can also be detected in patients with multiple sclerosis (MS).^8^ Indeed, retinal atrophy is associated with brain atrophy in MS, suggesting that both gray matter structures undergo similar degenerative processes.^9^ Thus, if structural changes in the retina parallel structural changes in the brain, molecular imaging of the retina is likely to be informative of the molecular basis of CNS damage in demyelinating diseases.

The aim of this study was to assess in vivo several molecules in the retina of patients with MS during the chronic phase or during the acute inflammation due to AON by Raman spectroscopy. As such, we built a prototype Raman spectroscopy device by adapting a scanning laser ophthalmoscope and coupling it to a spectrophotometer (Fig. 1). The Raman spectrophotometer was designed to obtain 80% of the signal from the retinal ganglion cell (RGC) layer, although other layers also contribute to the signal. We performed a hypothesis-based study by quantifying the levels of 10 relevant molecules associated with energy and axon biology (CytC, flavin adenine dinucleotide [FAD], NADH, *N*-acetyl-aspartate [NAA]), excitotoxicity (glutamate [Glu]), neuronal and synaptic damage (amyloid-beta [Aβ], τ and α-synuclein [SNCA]), and lipids with cell signaling activities (phosphatidyl-ethanolamine [PE] and PC).

**Figure 1.**
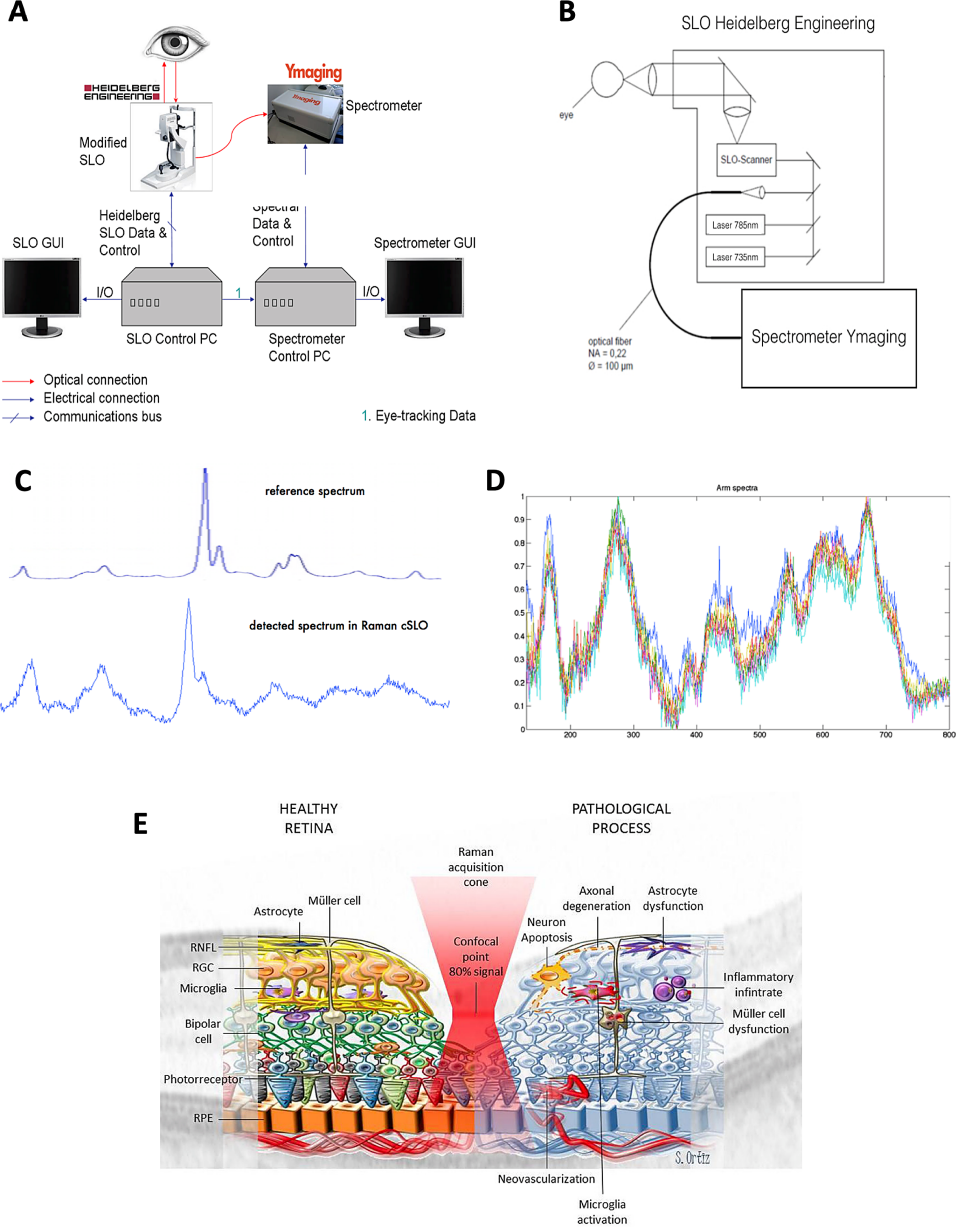
Prototype of the Raman spectrophotometer. (**A**) The device was an adapted Spectralis OCT device (Heidelberg Engineering). (**B**) The 785-nm laser from the device was substituted for a 785-nm laser from Andor Technology with an optic fiber, and the 735-nm laser was used for eye-tracking. (**C**) Raman spectra of ethanol obtained with the commercial Invia (Renishaw PLC. Wotton-under-Edge, UK) confocal Raman spectrophotometer compared with the confocal scanning laser ophthalmoscopy (cSLO)-Raman spectrophotometer prototype. (**D**) Reproducibility of the Raman signal from the retina of 10 healthy subjects. (**E**) Raman signal from healthy and inflamed retina. The confocal point of the prototype lies in the GCL, such that 80% of the signal comes from the GCL and its surrounding layers (retinal nerve fiber layer and inner plexiform layer), and the central retina (15°) was analyzed with a raster scan.

## Methods

### Study Design and Subjects

We conducted an observational study in prospective cohorts of patients with MS. The patients with MS studied here were part of the prospective MS-VisualPath cohort followed at the Hospital Clinic of the University of Barcelona, Spain. The cohort was set up in 2011, and it remains open and is described in detail elsewhere.^10^^,^^11^ For this analysis, we included 143 consecutive patients with MS fulfilling the 2010 McDonald criteria^12^ and with up to 5 years of follow-up. Changes in disease-modifying therapies were allowed following standard-of-care practices as described before.^11^ We also analyzed 15 consecutive patients with AON enrolled into the Barcelona prospective AON-VisualPath cohort at the Hospital Clinic of the University of Barcelona, Spain, from 2017 to 2019. This cohort is described elsewhere, including detailed ophthalmologic examination.^6^^,^^13^ Indeed, we analyzed 5 patients with NMO and anti–aquaporin-4 antibodies (NMO-AQP4) and 3 with myelin oligodendrocyte glicoprotein (MOG)-associated encephalitis (MOGAD) with anti-MOG antibodies, as described previously.^7^ Regarding the healthy controls, we collected 34 healthy volunteers matched for sex and age with the MS cohort as well as 12 healthy volunteers older than 55 years to analyze the effect of age on each of the molecules. Patients and controls were evaluated by the neuro-ophthalmologists (BS-D, AC-C, MD-B) and an optometrist (SA-A) to exclude underdiagnosed cases of ophthalmic diseases (e.g., glaucoma). Patients were also excluded if they had severe myopia (>–6.0 dioptres (dp) or axial eye length >26 mm), severe hypermetropia (>5 dp), cylinder >3 dp, optic nerve drusen, cataracts, current or previous glaucoma, lens opacities, previous surgery, or any other retinal disease, which may be relevant for the older control group. In addition, the neurology team (IP-V, SL, MS) ruled out other neurologic conditions that may influence the retina (neurodegenerative diseases, stroke, etc.) as well as systemic conditions such as diabetes, hyperlipidemia, or obesity. The study is reported following Strengthening the Reporting of Observational Studies in Epidemiology (STROBE) guidelines.

### Ethical Statement

The study was approved by the Hospital Clinical Ethical Committee, and the patients were recruited by their neurologist after providing their signed the informed consent.

### Clinical Outcomes

The clinical scales used to collect information on the patients with MS annually over the first 3 years and then on year 5 were the Expanded Disability Status Scale (EDSS)^14^ and low-contrast visual acuity using the Sloan 2.5% contrast cards (SL25).^15^ Patients with AON were assessed by high-contrast visual acuity using the Early Treatment Diabetic Retinopathy Study (ETDRS) plates, as well as using the SL25, and color vision was evaluated using the Hardy Rand and Rittler pseudoisochromatic plates at baseline and monthly until month 6, as described in Lampert et al.^16^ Patients with NMO-AQP4 and MOGAD were evaluated with the EDSS scale.

### Raman Spectroscopy of the Retina

Raman spectroscopy was conducted using a Raman spectrophotometer v1.0 (Ymaging SL, Barcelona, Spain). The system was developed by adapting a Spectralis OCT device (Heidelberg Engineering, Heidelberg, Germany), which was used to noninvasively capture molecular information of the retina in less than 2.5 minutes per eye (Fig. 1). The 785-nm laser from the OCT was substituted for another 785-nm laser with higher thermal stability, and the device was connected to a spectrophotometer (Andor Technology, Belfast, UK) with an optic fiber. The 735-nm laser of the OCT was used for eye-tracking. The Spanish Agency of Medicines and Medical Devices approved the device for human use (AEMPS ref. 497/14/EC). The prototype has the confocal point in the ganglion cell layer (GCL) so that 80% of the signal comes from the GCL and the surrounding layers (retinal nerve fiber layer [RNFL] and inner plexiform layer [IPL]) (Fig. 1).^5^ We used a black tarp covering the subject to avoid any external light during acquisition, without pupillary dilatation. We acquired five measurements (30 seconds each) from a 15° segment of the central retina in each eye. The quality of the signal was monitored during the acquisition, and the optometrist had the possibility of increasing the number of acquisitions to a maximum of eight measurements per eye if the signal-to-noise ratio was not sufficiently good to ensure a Raman signal of sufficient quality. The Raman spectroscopy results are taken from one randomly chosen eye of the controls. For patients with MS and NMO, we only used eyes with no previous optic neuritis, whereas we used only the eye with incident AON for the patients with AON.

The Raman spectra of the candidate molecules were defined as described previously.^5^ Briefly, CytC, FAD, NADH, NAA, Glu, Aβ, SNCA, PE, and PC were purchased from Sigma-Aldrich (St. Louis, MO, USA) and used without further modification. For the spectra of the molecules in solution, the powders were mixed with a small amount (one to two drops) of PBS (1×, pH 7.4) and imaged at this high concentration, with the exception of NADH, which was measured in Tris pH 7.5 due to its interactions with the phosphate in PBS.^17^ Eight different concentrations were prepared in PBS/Tris by dilution (4 M, 3 M, 2 M, 1 M, 0.5 M, 0.1 M, 0.05 M, and 0.01 M: pH 7). Three to five spectra were taken at each concentration.

### Optical Coherence Tomography

Retinal scans were performed using a Spectralis OCT device (Heyex 5.30; Heidelberg Engineering) under standard conditions and using the eye-tracking modality without pupillary dilatation. The peripapillary retinal thickness (pRNFL, µm) was measured using a ring scan of 12° diameter, automatically centered on the optic nerve head (automatic real time [ART] = 100; 1536 A-scans per B-scan). Macular layer thicknesses were evaluated using a 20 × 20-degree horizontal raster scan centered on the fovea, including 25 B-scans (ART ≥9; 512 A-scans per B-scan). Intraretinal layer segmentation was performed in a semiautomated fashion using the Spectralis segmentation algorithm (6.0c version) and a 6-mm ring area grid, with an optometrist manually correcting obvious errors blind to the conditions. We estimated the thicknesses (µm) of the RNFL, ganglion cell plus inner plexiform layer (GCIPL), inner nuclear layer, outer plexiform layer, outer nuclear layer (ONL), and photoreceptor layer (PRL). All spectral-domain OCT scans fulfilled OSCAR-IB criteria,^2^ and scans with a sub-standard signal-to-noise ratio or retinal thickness algorithm failure were repeated or the data were excluded.

### Statistical Analysis

All analyses were carried out using R software, first testing that the variables conformed to a normal distribution with the Shapiro–Wilks test. We described the baseline features of the study population and the clinical characteristics using the absolute and relative frequencies for categorical variables. For quantitative variables, we used medians and the interquartile range to describe the basal features of the study population. No imputation strategies were applied, and missing data were omitted. The percentage of missing data for independent variables did not exceed 7% in any of the data sets at each visit. Test–rest analysis was assessed through the relative error. A *t*-test, ANOVA, and pairwise comparisons were performed to compare the groups as appropriate, and correlations were analyzed using a Pearson correlation. Correction for multiple testing was done using false discovery rate with Benjamini–Hochberg procedure.

## Results

### The Retina of Healthy Controls and the Molecular Changes With Age and Sex

**Figure 2.**
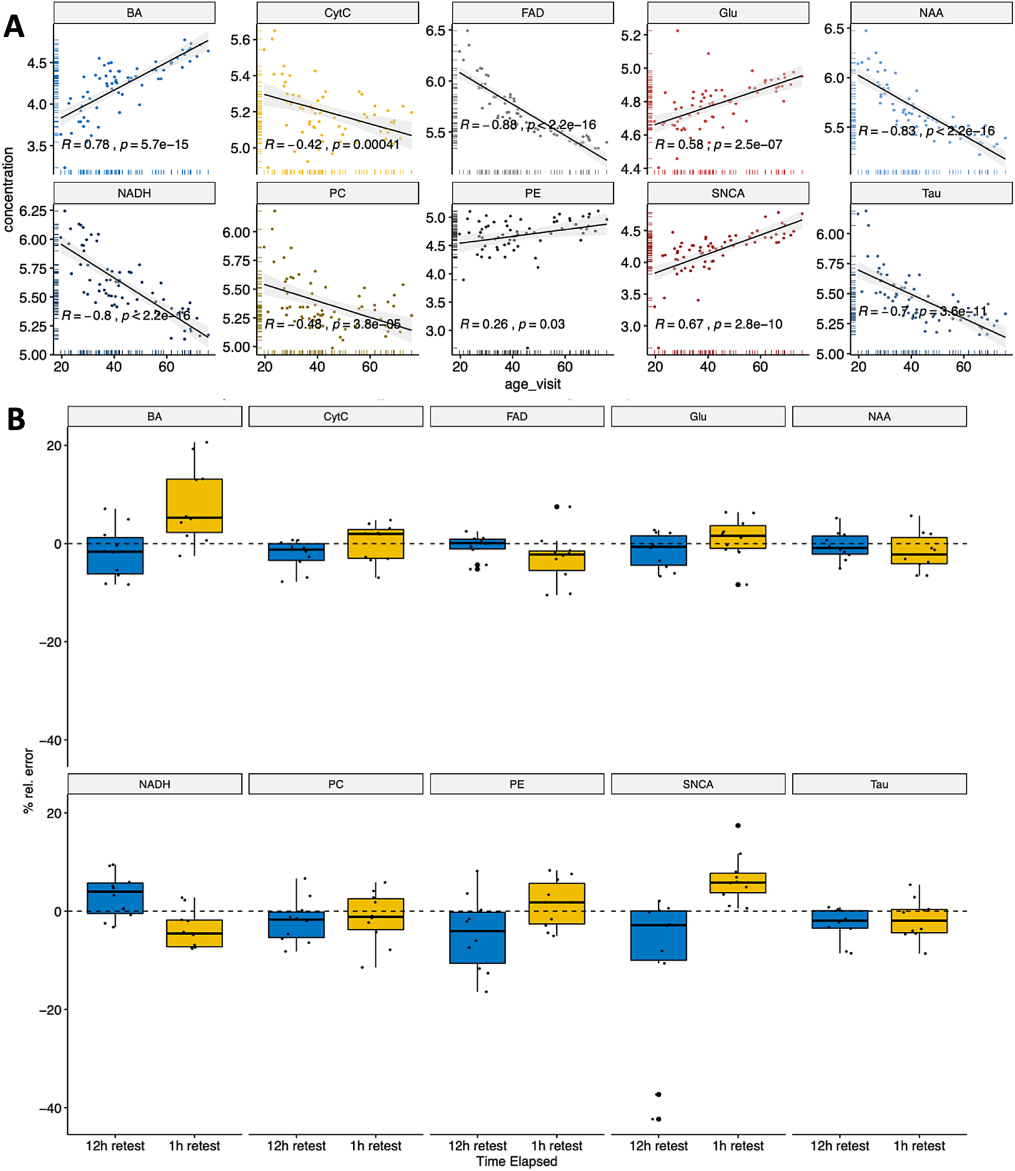
Retinal molecules in healthy controls measured by Raman spectroscopy. (**A**) Levels of the different proteins and age distribution: the scatterplots show the levels of CytC, FAD, Glu, NAA, NADH, PC, PE, Aβ, SNCA, and τ quantified by Raman spectroscopy on one randomly selected eye from healthy controls of different ages. The Pearson correlation *r* value and *P* value are shown. (**B**) Test–retest variability of the molecules in the retina at 1 and 12 hours (see Table 2 for numerical values). The results are shown as the relative error ([absolute difference/mean] * 100%).

We analyzed two groups of healthy controls (HCs, *n* = 68) to evaluate the influence of age on the levels of the molecules studied in the retina when assessed by Raman spectroscopy (Table 1). One group (*n* = 56) was composed of young adults matched with the relapsing-remitting multiple sclerosis (RRMS) group with a mean age of 38.2 years (range, 29.8–45.4), of whom 39 were females 39 (65%), while the other group (*n* = 12) was composed of older subjects with a mean age of 66.0 years (range, 61.8–68.4), of whom 8 were female (67%). We found that age significantly influenced the levels of all the molecules examined, with an increase in Glu, Aβ, SNCA, and PE and a decrease in CytC, FAD, NAA, NADH, PC, and τ with age (Fig. 2A). By contrast, we did not find a significant effect of sex on any of the molecules (Supplementary Fig. S1). Considering the effect of age in the levels of these molecules, an analysis by disease subtype was not performed due to the strong interaction between age and progressive disease.

**Table 1.** Clinical Characteristics of Patients With Demyelinating Diseases

Characteristic	MS	AON	NMO Anti–AQP-4^+^	MOGAD	Young HCs	Old HCs
*n*	143	15	5	3	34	12
Sex (female), *n* (%)	101 (70.62)	11 (73.33)	2 (40.0)	1 (33.0)	39 (64.83)	8 (66.66)
Age, years	43.5 [36.3–52.6]	31.4 [26.7–37.3]	42.8 [38.0–44.9]	53.8 [52.9–54.4]	38.2 [29.8–45.4]	66.0 [61.8–68.4]
Disease duration^*^	9.66 [5.50–15.8] years	15 [11–118] days	3.84 [0.99–5.38] years	1.54 [0.96–6.59] years	NA	NA
Subtype	RRMS: 124	Idiopathic: 5			NA	NA
	CIS: 7	MS: 10	NA	NA		
	PPMS: 9	•Relapse: 2				
	SPMS: 3	•First relapse: 8				
EDSS	1.5 [1–2]	NA	NA	NA	NA	NA
SL25^†^	23 [11.5–25.5]	ON: 0 [0–14.5]	NA	NA	NA	NA
		NON: 32 [28–33.5]				
DMT	Yes^‡^: 104	NA	NA		NA	NA
	No: 31			NA		
	NA: 8					

CIS, clinically isolated syndrome; DMT, disease modifyin therapy; NA, not available; ON, optic neuritis; NON, non–optic neuritis; PPMS, primary-progressive multiple sclerosis; RRMS, relapsing-remitting multiple sclerosis; SPMS, secondary-progressive multiple sclerosis.

* AON disease duration: days since the onset of AON.

† SL25 in patients with MS: mean of both eyes for NON eyes.

‡ Disease-modifying therapies in patients with MS, number of patients: fingolimod, 3; glatiramer acetate, 22; interferon beta1b, 21; interferon beta1b i.m., 15; interferon beta1a s.c., 23; natalizumab, 9; teriflunomide, 4; dimethyl-fumarate, 5.

In order to assess the test–retest consistency of the molecular quantification by Raman spectroscopy, we rescanned a subgroup of 6 HCs and 5 MS cases at 1 and 12 hours. We found that the molecules studied had a relatively low error in the 1-hour retest, in general less than 5%, except for Aβ and SNCA. Similarly, in the 12-hour retest, only PE and SNCA showed a relative error above 5%, with no differences between the HCs or patients with MS (Fig. 2B and Table 2).

**Table 2.** Test–Retest of the Molecules at 1 and 12 Hours in Controls (*n* = 11)

Molecule	1-h Retest	12-h Retest
Aβ	8.583 (7.373)	4.582 (2.999)
CytC	3.425 (1.528)	2.573 (2.728)
FAD	4.581 (3.750)	1.690 (1.775)
Glu	3.392 (2.738)	2.956 (2.273)
NAA	3.622 (2.079)	2.397 (1.646)
NADH	4.713 (2.442)	4.470 (3.110)
PC	4.277 (3.307)	3.941 (2.733)
PE	4.325 (2.460)	6.982 (5.324)
SNCA	6.568 (5.018)	10.700 (15.774)
τ	3.451 (2.662)	2.881 (3.150)

The results shown are the mean (SD) of relative error ([Absolute difference/mean] * 100%).

### Molecular Profiles in the Retina of Patients With MS

**Figure 3.**
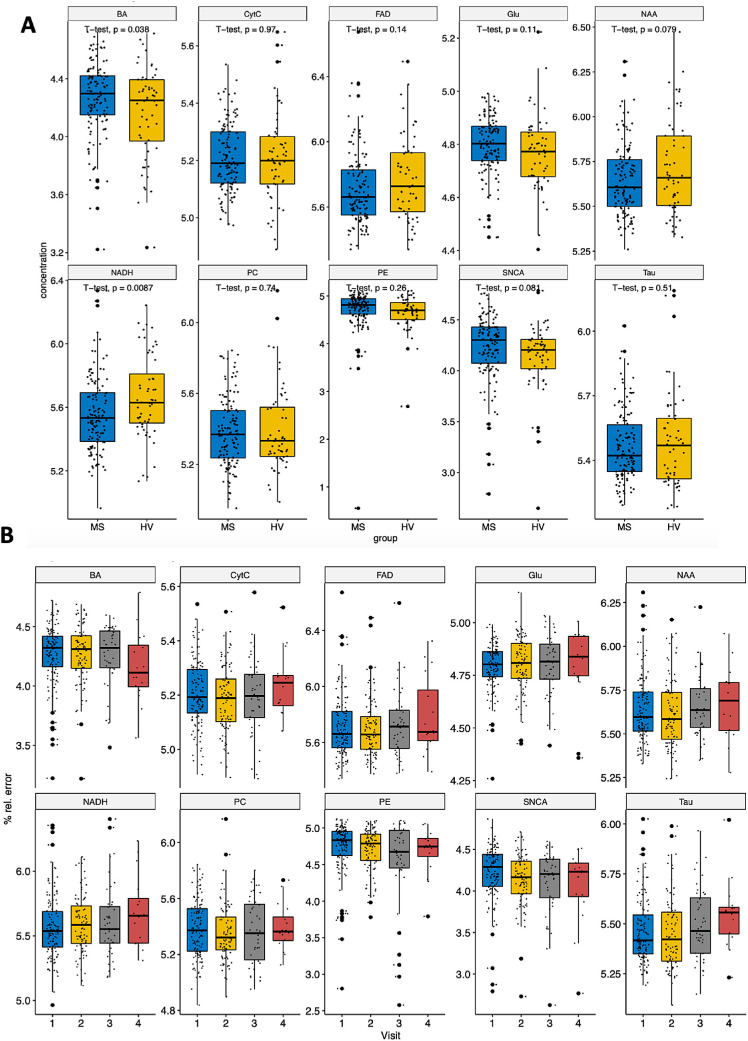
Differences in the retinal molecules in patients with MS. (**A**) Cross-sectional analysis. (**B**) Longitudinal analysis. Group comparisons were performed with a *t*-test and the time-series analyses were carried out with a repeated-measurements ANOVA.

To examine the changes in the candidate molecules due to retinal damage in patients with MS (Table 1), we first analyzed the differences during the remission phase relative to the HCs in a cross-sectional manner. There was a significant decrease in NADH levels in the retinas from patients with MS and a trend toward a decrease in NAA levels and an increase in Aβ levels relative to the controls (Fig. 3A). Indeed, we found that the NAA levels were correlated with 2.5% low-contrast vision (*r* = −0.36, unadjusted *P* = 0.027), although such correlation was not significant after correction for multiple testing (adjusted *P* = 0.27). In addition, we compared the changes in the candidate molecules in the retina of patients with MS with those in patients with NMO, with the latter associated with more severe CNS damage. Similar trends to those detected in patients with MS were observed in the retinas of patients with NMO-AQP4 and MOGAD, although they were not significant due to the small sample size of the NMO group (Supplementary Fig. S2). These results suggest that chronic retinal damage in demyelinating diseases is associated with a common metabolic profile.

We also analyzed the changes to the molecules in the retina of patients with MS over time in our prospective cohort. The longitudinal analysis in patients with MS was done on an annual basis, with a mean follow-up of 560 (0–784) days and a median of 2 (1–3) visits. We found a significant increase in NADH over time (unadjusted *P* = 0.011), although it was not significant after adjustment for multiple comparisons (adjusted *P* = 0.081) and a trend toward an increase in FAD (*P* = 0.06), whereas we observed a significant decrease in Aβ over time (unadjusted *P* = 0.012, adjusted *P* = 0.066; Fig. 3B).

Finally, we analyzed the relationship between structural and molecular changes in the retina, assessing the correlation between the levels of each molecule and the thickness of the retinal layers measured by OCT in patients with MS. We observed significant correlations between the thickness of the pRNFL and PE levels (*r* = 0.36, unadjusted *P* = 0.0026, adjusted *P* = 0.026); the thickness of the GCIPL and PE levels (*r* = 0.3, unadjusted *P* = 0.015, adjusted *P* = 0.15); the ONL thickness and beta-amiloid (BA) (*r* = −0.27, unadjusted *P* = 0.027, adjusted *P* = 0.11), PC (*r* = 0.24, unadjusted *P* = 0.056, adjusted *P* = 0.11), and SNCA (*r* = −0.22, unadjusted *P* = 0.075, adjusted *P* = 0.12); and the photoreceptor layer (PRL) thickness and FAD (*r* = 0.34, unadjusted *P* = 0.005, adjusted *P* = 0.024), PC (*r* = 0.35, unadjusted *P* = 0.0043, adjusted *P* = 0.024), SNCA (*r* = −0.3, unadjusted *P* = 0.016, adjusted *P* = 0.04), and τ (*r* = 0.31, *P* = 0.011, adjusted *P* = 0.038) (Supplementary Fig. S3). Therefore, damage to the retina in MS is associated with a molecular signature that is indicative of neuronal and synaptic damage.

### Changes to Retinal Molecules During Acute Inflammation in AON

**Figure 4.**
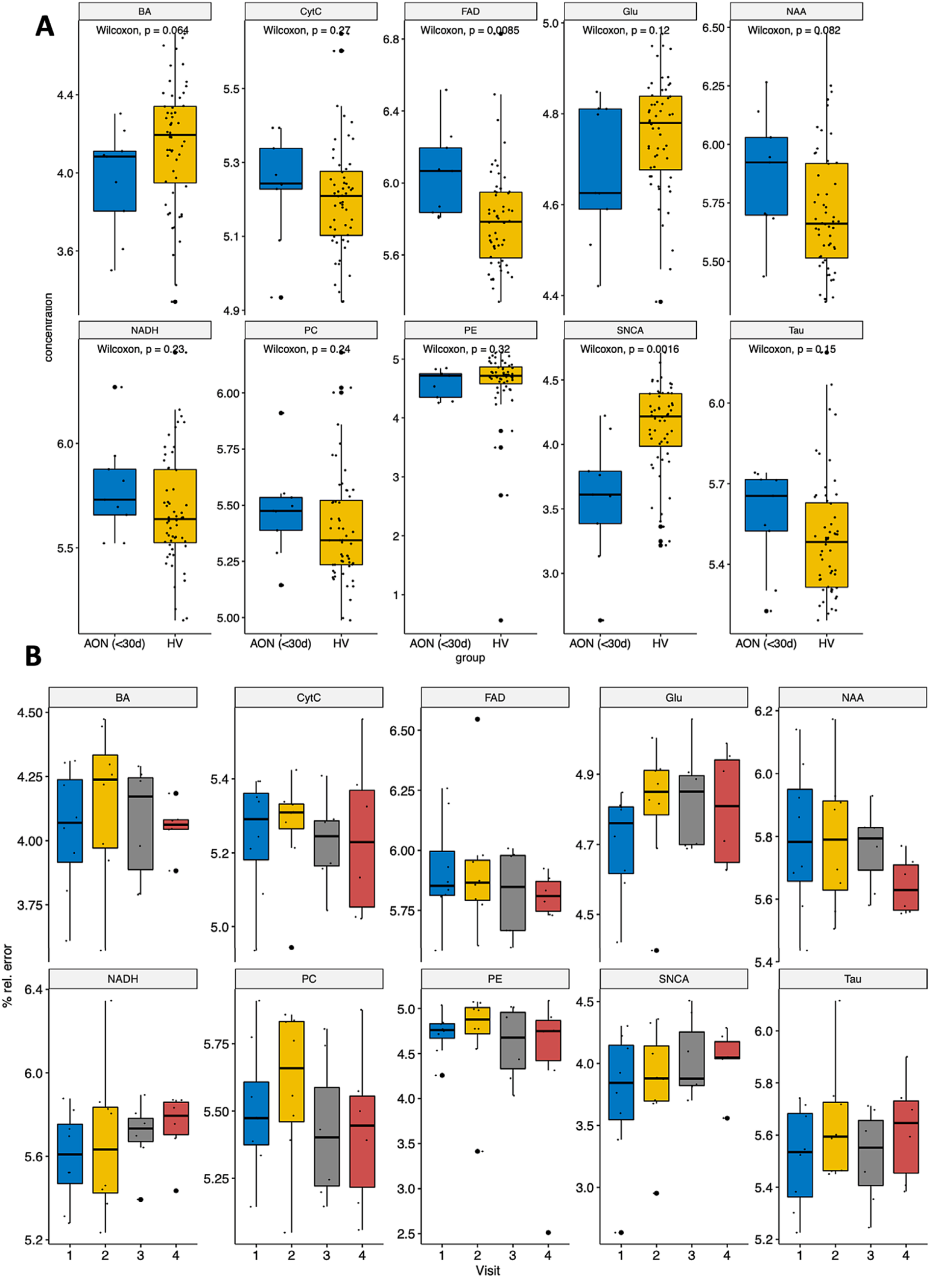
Differences in the retinal molecules in AON eyes. (**A**) Cross-sectional analysis. (**B**) Longitudinal analysis. Group comparisons were performed with a *t*-test and the time-series analyses were done with a repeated-measurements ANOVA.

To assess the changes in the metabolic profile of the retina during acute inflammation, we analyzed a prospective cohort of patients with AON both in the acute phase and longitudinally during recovery. We evaluated each of the molecules in the affected eye in the acute/inflammatory phase (baseline visit <30 days after onset) and after recovery (last visit >180 days after onset). In the acute phase, there was a significant increase in FAD (unadjusted *P* = 0.008, adjusted *P* = 0.042) in the affected retinas (AON eyes) and a decrease in SNCA levels (unadjusted *P* = 0.0016, adjusted *P* = 0.015) (Fig. 4A). No correlations between the levels of any of the molecules and visual acuity were found at baseline. When the longitudinal changes to these molecules were assessed by comparing their levels at baseline with those during the recovery process at months 2, 4, and 6 after AON onset, a significant increase in Glu was observed by month 6 (unadjusted *P* = 0.036, adjusted *P* = 0.24) (Fig. 4B). Finally, the relationship between the structural changes to the retina assessed by OCT and the molecular changes was evaluated by analyzing the correlation between the distinct molecules and the retina layer thickness in the acute disease phase. A significant correlation was detected between the pRNFL thickness and Glu levels (*r* = −0.62, unadjusted *P* = 0.025, adjusted *P* = 0.089), as well as for the GCIPL thickness and FAD levels (*r* = −0.62, unadjusted *P* = 0.025, adjusted *P* = 0.33) (Supplementary Fig. S4).

## Discussion

In this study, we found that there are changes in several key molecules in the retina of patients with acute or chronic damage due to neuroinflammation that are related to energy supply, as well as axon and synapse biology. In patients with a chronic inflammatory process due to MS, there is a loss of metabolites involved in energy production and axon degeneration in the retina, such as NADH and NAA,^18^ and these levels are correlated with scales of disability. Longitudinal assessment confirmed the chronic decrease of NADH and FAD, and the retinas from patients with MS had higher levels of Aβ that decreased over time. In association with acute inflammation due to AON, there was more FAD in the retina and less SNCA at disease onset, with Glu levels increasing during the recovery period. In addition, we found a correlation between retinal atrophy and the changes to several of the molecules tested, indicating a structural–molecular relationship in inflammatory CNS diseases.

The most prominent changes detected were in metabolites related to the mitochondria and energy supply (NADH, FAD), which are affected by both acute and chronic inflammatory damage to the retina.^19^^,^^20^ These results are consistent with the abundant literature describing the impairment of mitochondrial function, preferentially affecting complex I and IV in the brain of patients with MS,^21^ as well as altered mitochondrial transport along the axons.^22^ In addition, more NADH and less NAD^+^ have been described in the serum of patients with MS,^23^ probably secondary to the chronic oxidative stress observed in the brain of patients with MS. Moreover, after axonal transection due to inflammatory damage, axonal degeneration is regulated by several key molecules that influence NAD levels, such as nicotinamide nucleotide adenylyltransferase 2 (NMNAT2), sterile alpha and TIR motif 175 containing 1 (Sarm1), and phosphate starvation response 1 (PHR1),^18^ suggesting that NADH levels may also reflect the extent of axon damage.

Similarly, there was a decrease in another marker of axon function, NAA, in association with chronic inflammatory damage to the retina, and indeed, NAA abnormalities (including the NAA/myoinositol ratio) have been identified in the brain of patients with MS by MRS.^24^^,^^25^ Finally, the evolution of acute retinal inflammation was associated with an increase in glutamate, suggesting a role for excitotoxicity in the evolution of focal MS lesions, such as AON.^26^ Altered glutamate levels in the brain of patients with MS also have been observed by MRS,^27^^–^^29^ supporting a role for glutamate-mediated excitotoxicity in the evolution of MS.

Our study also identified alterations to several peptides/proteins related to the maintenance of neurons, axons and synapses, and neurodegeneration, such as Aβ, τ, and SNCA. Several amyloid-forming molecules are found in MS brain lesions, including alpha beta crystallin alpha B (CRYAB), amyloid precursor protein, major prion protein, and τ.^30^ Aβ levels decrease in the cerebrospinal fluid (CSF) of patients with MS in accordance with the pro- and anti-inflammatory cytokines detected.^31^^–^^33^ Indeed, the accumulations of Aβ aggregates and oligomers in the brain and CSF of patients with MS^34^ suggest Aβ may be implicated in the response to inflammatory damage to neurons and synapses. By using radioligands targeting Aβ for PET imaging studies (the PIB compound), a significant loss in the Aβ signal was seen in patients with MS, which may be related to either neuroaxonal damage or myelin loss.^35^ SNCA is known to modulate T-cell activation in the peripheral immune system and during CNS inflammation in the animal model of MS.^36^^,^^37^ Indeed, the immune response to another member of the synuclein family, β-synuclein, can trigger gray matter–specific autoimmune attack in animal models, and β-synuclein–specific T cells are enriched in progressive MS.^38^ SNCA is altered in the CSF of patients with MS,^39^^,^^40^ and its expression increases in active plaques within the brain,^41^ possibly an indicator of synaptic dysfunction, axon damage, and/or neuronal loss. Therefore, the alterations to Aβ and SNCA in the retina of patients with demyelinating disease may be surrogates of neuronal damage, and thus, they might represent biomarkers of CNS degeneration in neuroinflammatory diseases.

Our study has several limitations. First, the Raman signal (A-scans) was obtained from a cylinder across all retina layers with particular focus on the RGC layer, which constitutes about 80% of the signal.^5^ However, other layers also contribute, limiting the interpretation regarding the layer/cell specificity. In addition, the retinas were imaged using a raster scan covering the central retina, and the signal was averaged. For this reason, the results represent an analysis of a diffuse damage to the retina, the most commonly observed in MS,^42^ but they lose topographic definition. Therefore, the molecular information derived from this approach lacks cellular resolution, impeding the identification of the cellular sources of each metabolite. Second, we conducted a candidate analysis of 10 molecules previously identified as relevant to key pathways of CNS damage in MS, some of which were previously seen to be altered in association with retinal inflammation by Raman spectroscopy (CytC, NADH, NAA, and PC).^5^ Many other molecules may be altered in demyelinating disease, and some may be better biomarkers of the process. We selected the candidate approach to interrogate well-known biological features with this technology, yet future studies involving metabolomic screening should be able to identify other molecules and pathways altered in the retina of patients with demyelinating diseases. The signal-to-noise ratio was not very high with this prototype due to the small power of the Raman signal. However, our results are consistent with the literature, and the longitudinal analysis contributes to improve the reliability of the results. Future technological developments in this technology with enhanced ratios will improve the clinical usefulness of retina Raman spectroscopy. Considering the design as a pilot study, the results are provided as unadjusted *P* values as well as adjusted for multiple comparisons. This implies that validation in new cohorts will be required to confirm the findings not significant after multiple comparisons adjustment. In addition, standardization between Raman devices will be required before generalization of the results.^43^

In conclusion, this study indicates that acute and chronic inflammation of the retina induced by demyelinating diseases is associated with significant alterations in molecules related to energy metabolism and excitotoxicity, as well as with neuronal and synaptic maintenance. Considering that the retina is considered a gray matter structure of the CNS and given the association between retinal and CNS damage in patients with MS, the molecular changes observed here are likely to be representative of the overall molecular changes in the gray matter associated with demyelinating diseases.

## Supplementary Material

Supplement 1
