# Supplementary material for: In Vivo Molecular Changes in the Retina of Patients With Multiple Sclerosis
Source: Invest Ophthalmol Vis Sci. 2021 May 11;62(6):11. doi: 10.1167/iovs.62.6.11 (PMC8114005; doi:10.1167/iovs.62.6.11)
Supplement: Supplement 1 [file iovs-62-6-11_s001.pdf]

## Figure S1

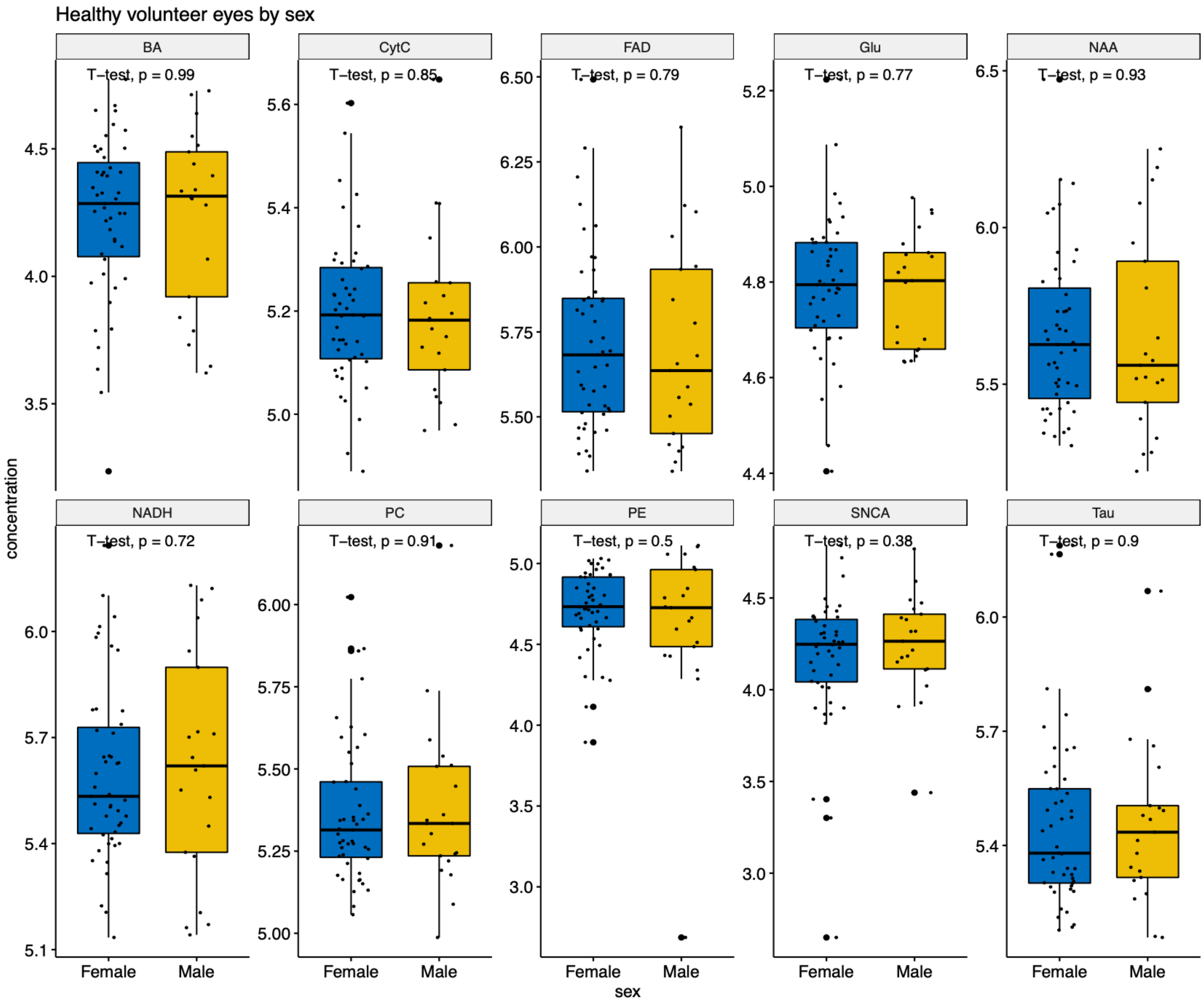
Healthy volunteer eyes by sex
BA
T–test, p = 0.99
CytC
T–test, p = 0.85
FAD
T–test, p = 0.79
Glu
T–test, p = 0.77
NAA
T–test, p = 0.93
NADH
T–test, p = 0.72
PC
T–test, p = 0.91
PE
T–test, p = 0.5
SNCA
T–test, p = 0.38
Tau
T–test, p = 0.9
concentration
Female
Male
sex

# Figure S2

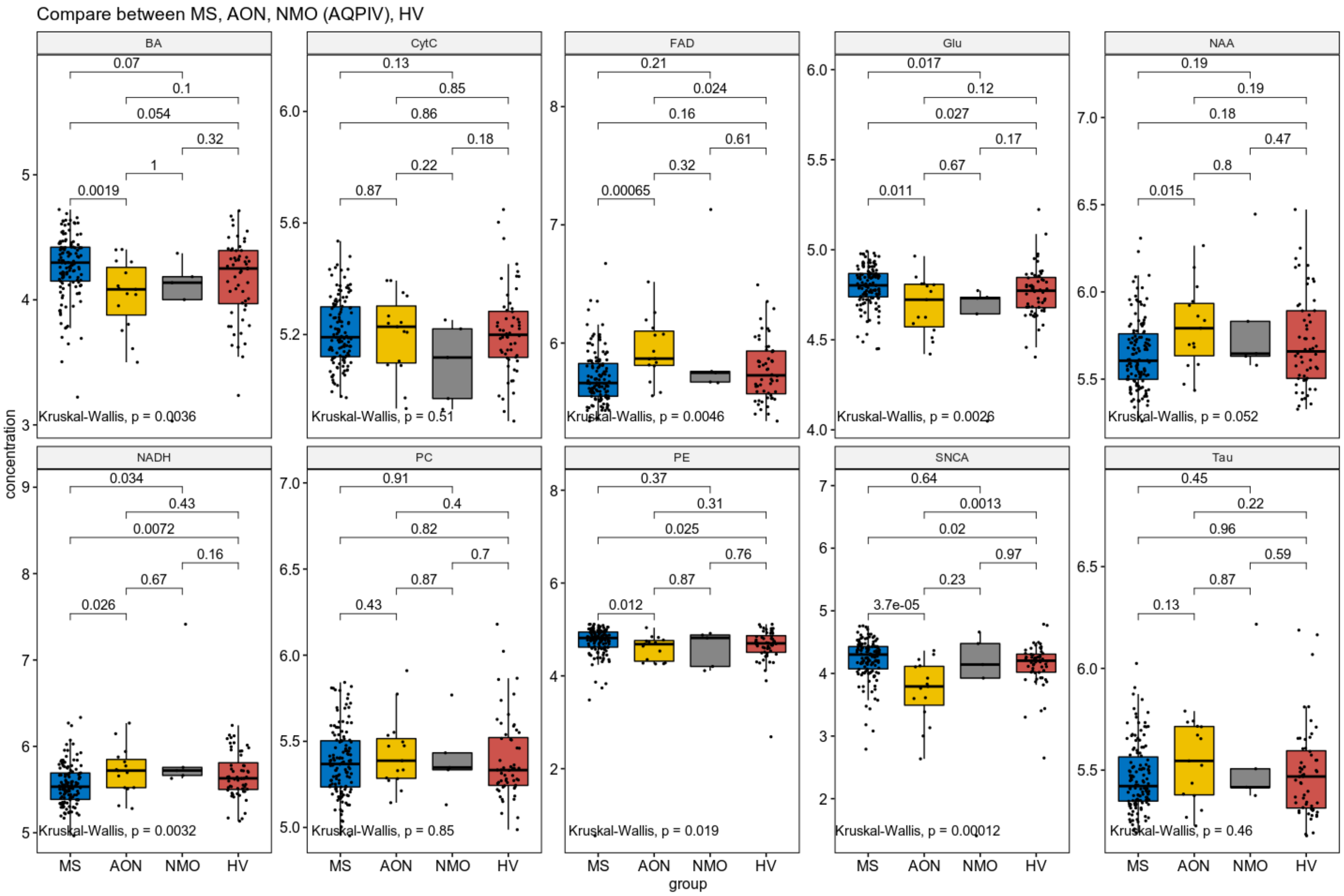

Compare between MS, AON, NMO (AQPIV), HV
BA
CytC
FAD
Glu
NAA
NADH
PC
PE
SNCA
Tau
concentration
group
MS
AON
NMO
HV
Kruskal-Wallis, p = 0.0036
Kruskal-Wallis, p = 0.51
Kruskal-Wallis, p = 0.0046
Kruskal-Wallis, p = 0.0026
Kruskal-Wallis, p = 0.052
Kruskal-Wallis, p = 0.0032
Kruskal-Wallis, p = 0.85
Kruskal-Wallis, p = 0.019
Kruskal-Wallis, p = 0.00012
Kruskal-Wallis, p = 0.46

# Figure S3

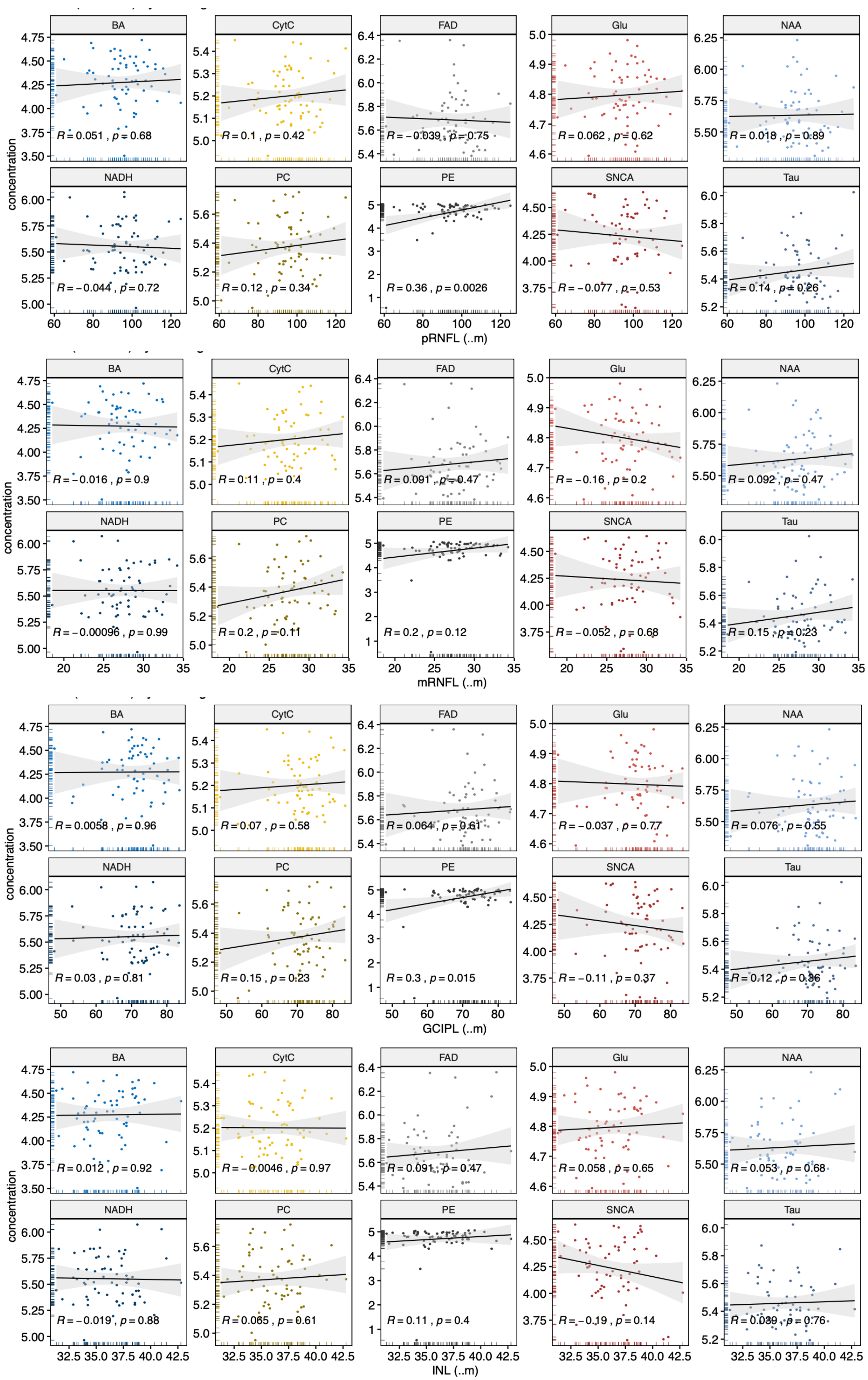
concentration
BA
R = 0.051 , p = 0.68
CytC
R = 0.1 , p = 0.42
FAD
R = −0.039 , p = 0.75
Glu
R = 0.062 , p = 0.62
NAA
R = 0.018 , p = 0.89
NADH
R = −0.044 , p = 0.72
PC
R = 0.12 , p = 0.34
PE
R = 0.36 , p = 0.0026
SNCA
R = −0.077 , p = 0.53
Tau
R = 0.14 , p = 0.26
pRNFL (..m)
concentration
BA
R = −0.016 , p = 0.9
CytC
R = 0.11 , p = 0.4
FAD
R = 0.091 , p = 0.47
Glu
R = −0.16 , p = 0.2
NAA
R = 0.092 , p = 0.47
NADH
R = −0.00096 , p = 0.99
PC
R = 0.2 , p = 0.11
PE
R = 0.2 , p = 0.12
SNCA
R = −0.052 , p = 0.68
Tau
R = 0.15 , p = 0.23
mRNFL (..m)
concentration
BA
R = 0.0058 , p = 0.96
CytC
R = 0.07 , p = 0.58
FAD
R = 0.064 , p = 0.61
Glu
R = −0.037 , p = 0.77
NAA
R = 0.076 , p = 0.55
NADH
R = 0.03 , p = 0.81
PC
R = 0.15 , p = 0.23
PE
R = 0.3 , p = 0.015
SNCA
R = −0.11 , p = 0.37
Tau
R = 0.12 , p = 0.36
GCIPL (..m)
concentration
BA
R = 0.012 , p = 0.92
CytC
R = −0.0046 , p = 0.97
FAD
R = 0.091 , p = 0.47
Glu
R = 0.058 , p = 0.65
NAA
R = 0.053 , p = 0.68
NADH
R = −0.019 , p = 0.88
PC
R = 0.065 , p = 0.61
PE
R = 0.11 , p = 0.4
SNCA
R = −0.19 , p = 0.14
Tau
R = 0.039 , p = 0.76
INL (..m)

# Figure S3 cont.

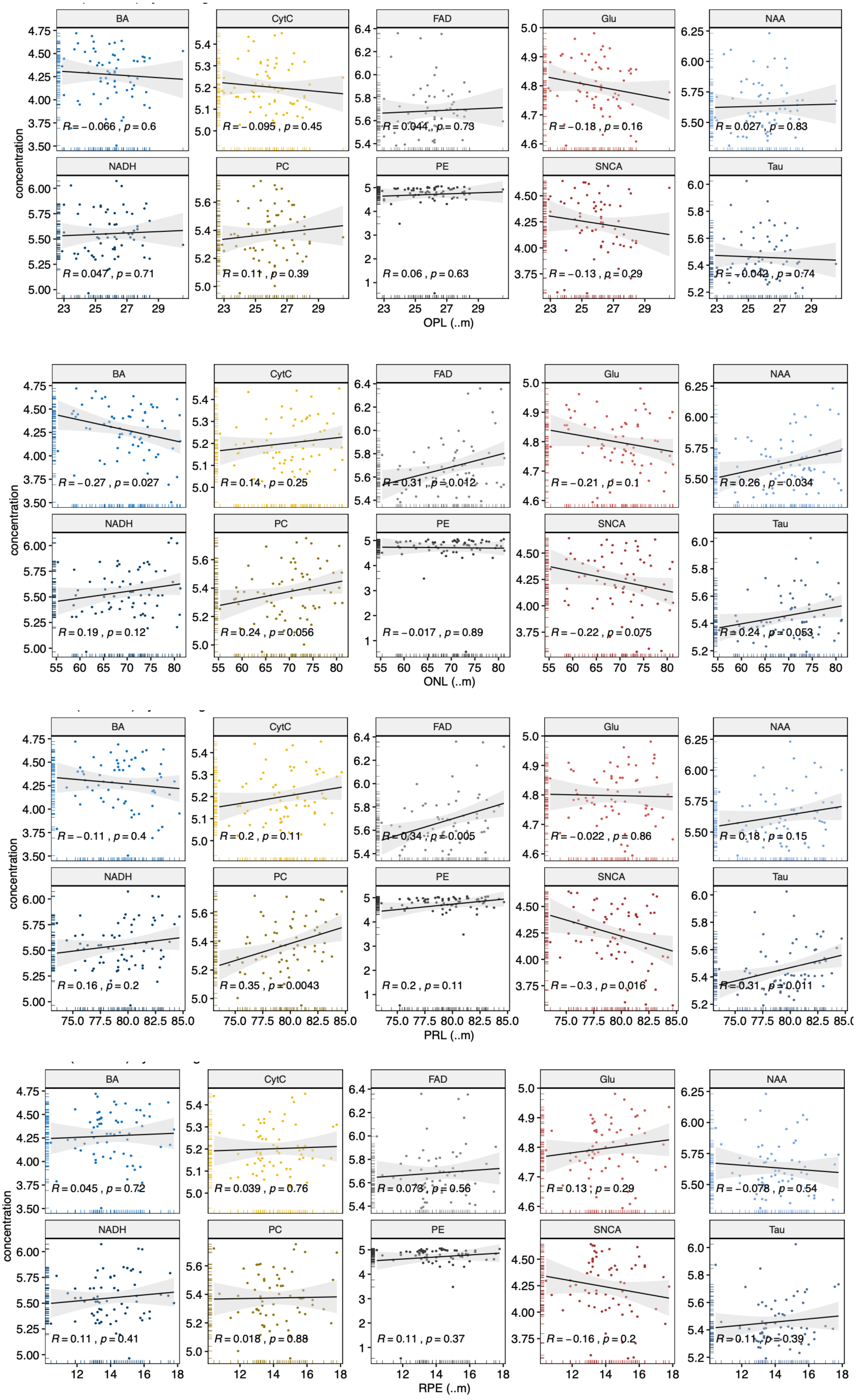

# Figure S4

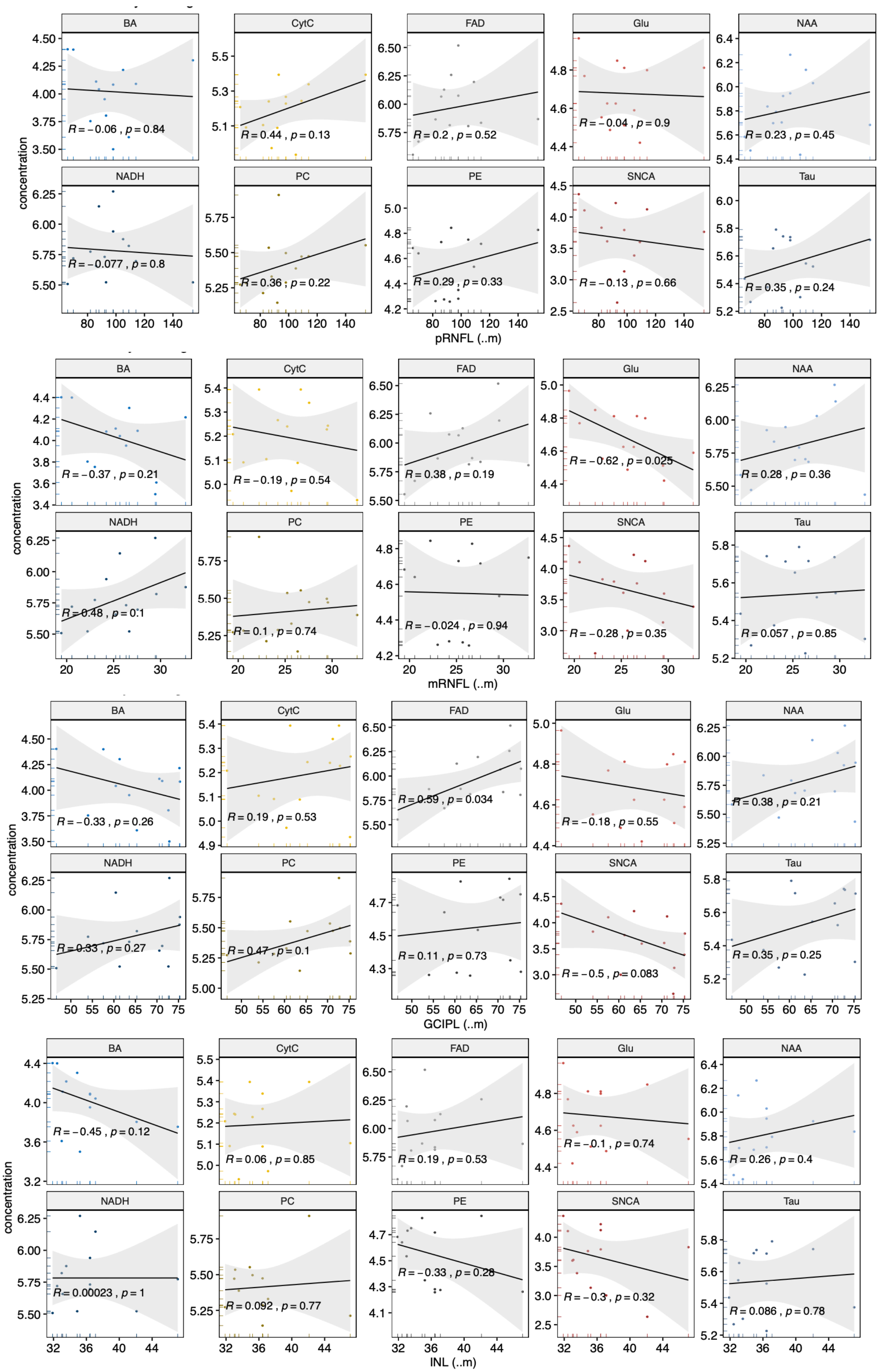

BA
CytC
FAD
Glu
NAA
NADH
PC
PE
SNCA
Tau
concentration
R = −0.06 , p = 0.84
R = 0.44 , p = 0.13
R = 0.2 , p = 0.52
R = −0.04 , p = 0.9
R = 0.23 , p = 0.45
R = −0.077 , p = 0.8
R = 0.36 , p = 0.22
R = 0.29 , p = 0.33
R = −0.13 , p = 0.66
R = 0.35 , p = 0.24
pRNFL (..m)
R = −0.37 , p = 0.21
R = −0.19 , p = 0.54
R = 0.38 , p = 0.19
R = −0.62 , p = 0.025
R = 0.28 , p = 0.36
R = 0.48 , p = 0.1
R = 0.1 , p = 0.74
R = −0.024 , p = 0.94
R = −0.28 , p = 0.35
R = 0.057 , p = 0.85
mRNFL (..m)
R = −0.33 , p = 0.26
R = 0.19 , p = 0.53
R = 0.59 , p = 0.034
R = −0.18 , p = 0.55
R = 0.38 , p = 0.21
R = 0.33 , p = 0.27
R = 0.47 , p = 0.1
R = 0.11 , p = 0.73
R = −0.5 , p = 0.083
R = 0.35 , p = 0.25
GCIPL (..m)
R = −0.45 , p = 0.12
R = 0.06 , p = 0.85
R = 0.19 , p = 0.53
R = −0.1 , p = 0.74
R = 0.26 , p = 0.4
R = 0.00023 , p = 1
R = 0.092 , p = 0.77
R = −0.33 , p = 0.28
R = −0.3 , p = 0.32
R = 0.086 , p = 0.78
INL (..m)

**Figure S4 cont.**

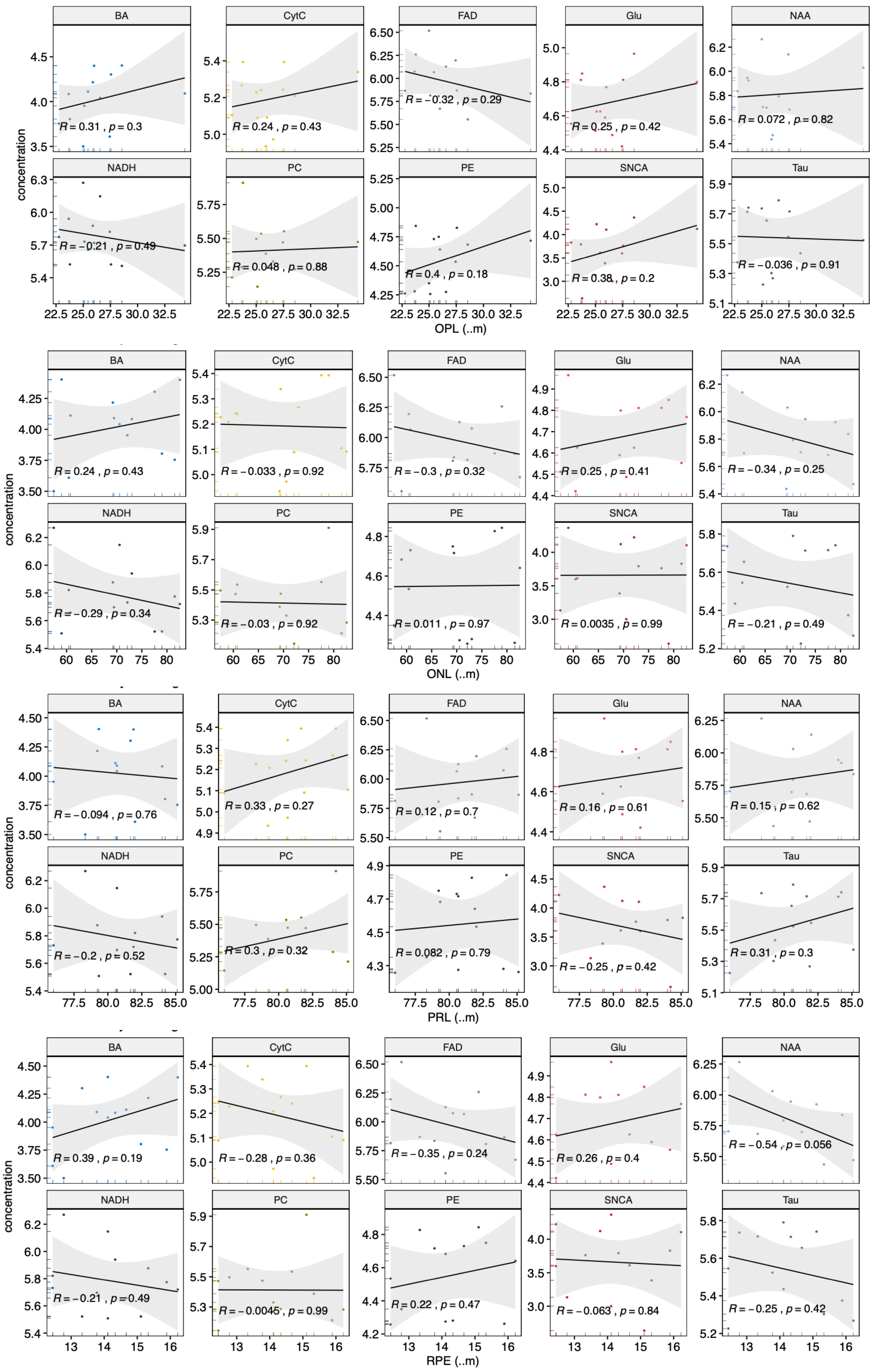

Figure S1. Effect of sex on the retinal molecules

Figure S2. Differences in the molecules between the MS, NMO-AQP4, AON and Healthy Control retinas

Figure S3. Correlation between retinal layer thickness and the levels of the molecules in MS patients

Figure S4. Correlation between retinal layer thickness and molecular levels in AON patients
